# Knowledge, Practices, and Attitudes Regarding Antimicrobial Resistance and Antibiotic Use Among Hospital Staff: Findings from a Two-Round Cross-Sectional Survey in an Italian Tertiary Care Hospital

**DOI:** 10.3390/antibiotics15080792

**Published:** 2026-08-16

**Authors:** Daniele Mengato, Elisabetta Conte, Giacomo Berti, Roberto Brunoro, Sara Lo Menzo, Margherita Boschetto, Elisabetta Lolli, Cristina Contessa, Maria Mazzitelli, Stefano Carraro, Ignazio Castagliuolo, Daniele Donà, Tiziano Martello, Annamaria Cattelan, Francesca Venturini, Vincenzo Baldo

**Affiliations:** 1 Hospital Pharmacy Unit, University Hospital of Padua, 35128 Padua, Italy; francesca.venturini@aopd.veneto.it; 2 Department of Cardiac, Thoracic, Vascular Sciences, and Public Health, University of Padua, 35131 Padua, Italy; elisabetta.conte.2@studenti.unipd.it (E.C.); vincenzo.baldo@unipd.it (V.B.); 3 Clinical Research and Projects Unit, University Hospital of Padua, 35128 Padua, Italy; giacomo.berti@aopd.veneto.it; 4 Department of Pharmaceutical Science, University of Milan, 20122 Milano, Italy; roberto.brunoro@unimi.it; 5 Infectious and Tropical Diseases Unit, University Hospital of Padua, 35128 Padua, Italy; sara.lomenzo@aopd.veneto.it (S.L.M.); mmazzitelli88@gmail.com (M.M.); annamaria.cattelan@aopd.veneto.it (A.C.); 6 Infection Control Division, University Hospital of Padua, 35128 Padua, Italy; margherita.boschetto@gmail.com (M.B.); elisabetta.lolli@aopd.veneto.it (E.L.); 7 Department of Directional Hospital Management, University Hospital of Padua, 35128 Padua, Italy; cristina.contessa@aopd.veneto.it (C.C.); tiziano.martello@aopd.veneto.it (T.M.); 8 Department of Safety and Bioethics, Section of Infectious Diseases, Università Cattolica del Sacro Cuore, 00168 Rome, Italy; 9 Department of Medical and Surgical Sciences, Fondazione Policlinico Universitario Agostino Gemelli IRCCS, 00168 Rome, Italy; 10 Microbiology and Virology Diagnostic Unit, University Hospital of Padua, 35128 Padua, Italy; stefano.carraro@aopd.veneto.it (S.C.); ignazio.castagliuolo@aopd.veneto.it (I.C.); 11 Division of Paediatric Infectious Diseases, Department of Women’s and Children’s Health, University of Padova, 35141 Padua, Italy; daniele.dona@unipd.it; 12 Preventive Medicine and Risk Assessment Unit, University Hospital of Padua, 35128 Padua, Italy

**Keywords:** antimicrobial resistance, antimicrobial stewardship, healthcare personnel, infection prevention and control, professional training, knowledge, attitudes and practices, repeated cross-sectional survey

## Abstract

**Background**: Education is a cornerstone of antimicrobial stewardship, yet evidence on its impact across the entire hospital workforce remains limited. This study aimed to assess knowledge, attitudes, and practices regarding antibiotic use and AMR among workers employed at an Italian tertiary-care hospital, and to explore the association between questionnaire performance and participation in antibiotic-related training activities. **Methods**: A repeated cross-sectional survey was conducted in 2024 and 2025 at a large Italian tertiary-care hospital (Padua, Northern Italy). A structured questionnaire adapted from an ECDC-validated instrument was distributed through the institutional email system to all hospital employees, including healthcare professionals and non-healthcare workers. The questionnaire investigated demographic and professional characteristics, participation in antibiotic-related training activities, and knowledge, attitudes and practices regarding antibiotic use and AMR. Questionnaire performance was assessed using both a binary scoring system and a partial credit scoring system. Multivariable linear regression analysis was performed to identify independent predictors of performance. **Results**: A total of 3193 participants completed the survey (1928 and 1265 in 2024 and in 2025, respectively). The mean (SD) total score was 5.75 ± 2.25 out of 15 points, with the lowest performance observed in the practice domain. No significant differences were found between the two survey years (2024 and 2025). Participants who attended antibiotic-related training performed significantly better than untrained participants (6.23 vs. 5.01; *p* < 0.001), with prior training emerging as the strongest independent predictor of questionnaire performance (β = 4.8; *p* < 0.001). Younger age was associated with better performance, while longer seniority (>30 years) was associated with lower scores. Among healthcare professionals, modestly higher scores were observed in 2025 compared with 2024, particularly in the practice domain. **Conclusions**: Persistent opportunities for improvement remain in AMR-related knowledge, attitudes and particularly practices across the hospital workforce. Participation in antibiotic-related training activities was associated with improved questionnaire performance, supporting the role of structured and continuous educational interventions within hospital AMS programs. Repeated institutional educational initiatives may contribute to strengthening stewardship-related awareness and practices over time.

## 1. Introduction

Antimicrobial Resistance (AMR) is recognized by the World Health Organization (WHO) as one of the major global public health threats [1] and has been described as a potential future pandemic threat [2]. According to a recent systematic analysis of the Global Burden of Disease, an estimated 4.71 million deaths were associated with bacterial AMR worldwide in 2021 [3]. AMR occurs when microorganisms no longer respond to antimicrobial medicines. As a result of drug resistance, antibiotics and other antimicrobial medicines become less effective, making infections increasingly difficult to treat and increasing the risk of disease spread, severe illness, disability and death [1]. The misuse and overuse of antimicrobials in humans, animals and plants are among the main drivers of selective pressure and of the dissemination of resistant pathogens [1]. Within healthcare settings, healthcare-associated infections (HAIs) and AMR are closely interconnected. Patients with HAIs frequently require broad-spectrum antimicrobial treatments because of the increased risk of multidrug-resistant organisms, contributing to further antimicrobial selective pressure and resistance development. Consequently, infection prevention and control (IPC) measures aimed at reducing HAIs also represent a key strategy for limiting antimicrobial use and slowing the emergence and spread of AMR [4]. In the wide context of stewardship activities, improving knowledge and awareness regarding appropriate antimicrobial use is therefore considered a key component of strategies aimed at controlling AMR, in association with changes in prescription policies [5,6]. Consistently, the WHO identifies education and training as essential elements of both antimicrobial stewardship (AMS) and IPC programmes, and recommends structured and continuous educational interventions to strengthen healthcare workers’ competencies and to promote appropriate antimicrobial use and IPC practices [7,8]. Consistently, the Italian National Action Plan on Antimicrobial Resistance (PNCAR 2022–2025) also identified antimicrobial stewardship, IPC, and the strengthening of education and training initiatives among healthcare professionals and the general population as key strategic actions to address AMR through a One Health approach [9]. Educational interventions have indeed been associated with improvements in antimicrobial stewardship knowledge and prescribing behaviours among healthcare professionals [10]. Assessing baseline knowledge and awareness of antimicrobial use and AMR represents a fundamental step for identifying educational needs, guiding targeted training, and evaluating its impact over time, particularly in hospital settings. Structured questionnaires have been widely applied to investigate knowledge gaps and factors associated with appropriate antimicrobial use, both among healthcare workers and in the general population, including large multi-country surveys coordinated by the ECDC [11,12,13,14,15,16,17]. However, evidence regarding AMR-related knowledge among the overall hospital workforce, including non-healthcare personnel, remains limited. In the Italian setting, published KAP studies have so far focused on selected professional categories, mainly physicians and nurses [12,18,19,20], and repeated cross-sectional assessments evaluating the association between institutional training exposure and questionnaire performance across the whole hospital workforce are, to our knowledge, still lacking.

Against this background, the present study used a structured questionnaire to assess knowledge, attitudes and practices regarding antibiotic use and AMR among healthcare and non-healthcare workers employed at an Italian tertiary care hospital, through two independent cross-sectional surveys conducted one year apart. The study also explored whether questionnaire performance differed according to self-reported participation in training activities on healthcare-associated infections and AMR, and across the two survey rounds, which took place during the implementation of institutional educational programmes.

## 2. Results

### 2.1. Study Population

A total of 3193 participants completed the survey across the two waves, with 1928 responders in 2024 and 1265 in 2025 (Table 1). The overall sample was predominantly composed of nurses (44%), followed by physicians (16%), HCAs (Health Care Assistants) (13%), and administrative staff (10%). Most participants were aged ≥ 55 years (33%) or 45–54 years (27%), with younger age groups (≤34 and 35–44 years) each accounting for approximately 20% of the sample. Regarding seniority, 42% had ≤10 years of service, while 22% had more than 30 years. Overall, 61% of respondents reported having attended at least one antibiotic-related training course (either ECM participation or completion of a hospital infection prevention course). The two survey waves were broadly comparable in terms of demographic and professional characteristics, although a slightly higher proportion of participants reported prior antibiotic-related training in 2025 compared with 2024 (64% vs. 58%).

### 2.2. Questionnaire Performance

Using the binary scoring system, participants achieved a mean total score of 5.75 ± 2.25 out of 15, with mean domain scores of 2.93 ± 1.48 for knowledge, 2.15 ± 1.05 for attitudes, and 0.68 ± 0.65 for practices (Table 2). No significant differences were observed between the 2024 and 2025 survey waves. Participants with prior antibiotic-related training consistently scored higher than untrained participants across the total score and all domains (all *p* < 0.001). Performance differed significantly by age for the total, knowledge, and attitude scores, with younger participants achieving higher scores than older groups, whereas practice scores did not vary significantly by age. Seniority at the hospital showed a heterogeneous association with performance.

Multivariable linear regression confirmed that prior training was the strongest independent predictor of questionnaire performance (β = 4.8, 95% CI 3.8–5.8; *p* < 0.001), with 5.0 points higher on knowledge, 3.6 on attitudes, and 6.5 on practices (all *p* < 0.001) after adjusting for survey year, profession, and seniority (Table 3). Non-healthcare staff scored consistently lower than healthcare staff across every domain (total: −12.0, 95% CI −13.0–−11.0; knowledge: −16.0; attitudes: −7.3; practices: −10.0; all *p* < 0.001). Greater seniority was associated with lower knowledge scores (>30 years: −6.4, 95% CI −8.2–−4.5, *p* < 0.001; 11–20 years: −2.6, *p* = 0.008; 21–30 years: −2.3, *p* = 0.020) and, to a lesser extent, lower total and attitude scores among staff with >30 years of seniority (*p* < 0.001), while practice scores were not significantly affected by seniority. Survey year (2025 vs. 2024) was not associated with total, knowledge, or attitude scores, but was associated with a modest improvement in practice scores (+2.3, 95% CI 0.4–4.1, *p* = 0.017).

### 2.3. Sensitivity Analysis: Partial Credit Scoring System

A sensitivity analysis using a partial credit scoring system (1 point for correct answers, 0.5 for directionally consistent responses, 0 for incorrect or “I don’t know” responses) yielded higher absolute scores compared to the binary approach (Table S3). The mean total score was 7.89 ± 2.38 out of 15 points, with domain-specific scores of 3.58 ± 1.38 for knowledge, 3.10 ± 1.01 for attitudes, and 1.20 ± 0.84 for practices. The partial credit system confirmed the primary findings: no significant differences between survey years (2024: 7.87 ± 2.34 vs. 2025: 7.91 ± 2.43; *p* > 0.05), but consistently higher scores among trained participants (8.49 ± 1.92 vs. 6.95 ± 2.68; *p* < 0.001).

### 2.4. Subgroup Analysis: Healthcare Professionals Only

When restricting the analysis to clinical healthcare professionals (*n* = 2730; 61% from 2024, 39% from 2025), excluding administrative and non-clinical staff, performance scores were modestly higher than in the full sample (Table S4). Mean total score was 6.05 ± 2.09 (vs. 5.75 ± 2.25 in the full sample), driven primarily by higher knowledge scores (3.09 ± 1.41 vs. 2.93 ± 1.48) and practice scores (0.75 ± 0.81 vs. 0.68 ± 0.80) (Table 4). In contrast with the overall sample, healthcare professionals showed a small but statistically significant improvement between survey years: total score increased from 5.99 ± 2.10 in 2024 to 6.16 ± 2.06 in 2025 (*p* = 0.046), with the most notable change occurring in the practice domain (0.71 ± 0.80 vs. 0.80 ± 0.82; *p* = 0.003). Participants with prior antibiotic-related training achieved significantly higher total and domain-specific scores than untrained participants (Table 4). Trained staff scored 6.27 ± 2.01 versus 5.59 ± 2.17 among untrained peers (*p* < 0.001), with significant differences across all three domains. Notably, a higher proportion of the 2025 cohort reported prior training compared to 2024 (74% vs. 65%; *p* < 0.001), potentially contributing to the observed temporal improvement in scores. Age and seniority patterns in the healthcare subgroup mirrored those of the full sample. Younger participants (≤34 years: 6.36 ± 1.96) consistently outperformed older groups (≥55 years: 5.81 ± 2.15; *p* < 0.001), with the gradient particularly evident in knowledge. Among seniority groups, those with ≤10 years of service ranked highest (6.24 ± 2.07), with performance declining progressively among those with >30 years (5.72 ± 2.09; *p* < 0.001).

## 3. Discussion

This repeated cross-sectional survey explored knowledge, practices, and attitudes regarding antibiotic use and AMR among employees of a large Italian tertiary-care hospital across two rounds (2024–2025) during the implementation of institutional antimicrobial stewardship and infection prevention and control educational activities. Overall, the results suggest that there may still be opportunities to further strengthen awareness of appropriate antibiotic use and its application in routine clinical practice. The mean binary total score, corresponding to approximately 38% of the maximum attainable, indicates that areas for improvement remain in AMR-related knowledge and stewardship-related practice. Scores were lowest in the practice domain (0.68/3), the area most closely related to the translation of knowledge into clinical decision-making. This finding supports the notion that awareness and theoretical knowledge, although essential, may not be sufficient on their own to promote appropriate clinical behaviour. Our findings therefore reinforce the concept that antimicrobial stewardship should not be regarded solely as a prescribing intervention, but also as a continuous educational process aimed at translating knowledge into everyday clinical decision-making. From this perspective, periodic assessment of AMR-related knowledge may represent a useful institutional tool to identify educational priorities and monitor stewardship implementation over time.

This dissociation between knowledge and practice is consistent with the previous antimicrobial stewardship literature. Davey et al. (2017) showed that knowledge-based interventions alone may be insufficient to sustainably change prescribing behaviour, while Dryden et al. (2017) emphasized that stewardship approaches must be translated into continuous, context-specific actions rather than isolated educational initiatives [21,22]. Similarly, recent systematic evidence has reported substantial variability in healthcare professionals’ adherence to infection prevention and antimicrobial stewardship practices, as well as ongoing concerns regarding inappropriate antibiotic prescribing [23]. The partial credit sensitivity analysis provided a more granular picture across the knowledge-to-practice spectrum and confirmed the primary findings. Participation in antibiotic-related training was the strongest independent predictor of performance across all domains (adjusted β = 4.8, *p* < 0.001). Although causality cannot be established from a cross-sectional design, the magnitude and consistency of this association across all analytical approaches, including the partial credit sensitivity analysis, suggest that structured educational activities represent one of the most actionable components of hospital AMS programmes.

Although no substantial differences between survey waves were observed in the overall sample, healthcare professionals showed modestly higher performance in the second survey round, particularly in the practice domain, in parallel with a higher self-reported training exposure. This temporal pattern is consistent with the hypothesis that repeated institutional educational activities may progressively strengthen stewardship-related competencies among clinical staff, although the repeated cross-sectional design of the study does not allow this inference to be tested at the individual level. Notably, 39% of respondents, including approximately 26% of clinical healthcare professionals, reported no prior participation in antibiotic-related training activities, suggesting that opportunities remain to further expand the reach of institutional educational initiatives. Integrating antibiotic-related training into onboarding pathways for newly recruited staff and reinforcing it through periodic refresher activities for all professional roles and seniority levels may represent a feasible strategy in this direction. An additional strength of the present survey is the inclusion of the entire hospital workforce rather than prescribers alone. Because AMS increasingly relies on multidisciplinary collaboration, involving physicians, nurses, pharmacists, microbiologists, infection prevention specialists, healthcare assistants, and administrative personnel, hospital-wide educational strategies should target the entire healthcare ecosystem, recognizing that appropriate antimicrobial use is influenced by multiple professional roles beyond prescribing itself. These considerations align with the international consensus on training as a core element of hospital AMS programmes and with the Veneto Region’s ARCO Project findings, which identified uneven training implementation as a key gap in regional AMS governance [24,25]. The seniority-stratified analysis revealed a nuanced and practically important pattern. Knowledge scores tended to be lower among staff with longer seniority, with significant deficits already evident among staff with 11–20 years of service (β = –2.6, 95% CI –4.6 to –0.69, *p* = 0.008) and becoming more pronounced beyond 30 years (β = –6.4, 95% CI –8.2 to –4.5, *p* < 0.001), whereas total and attitude scores were significantly lower only in the most senior group (>30 years). Notably, seniority was not a significant independent predictor of practice scores in the multivariable regression model, suggesting that the erosion of theoretical knowledge over time is not mirrored by a corresponding decline in day-to-day clinical behaviour; this dissociation may reflect the influence of institutional protocols, peer-driven learning, and accumulated clinical experience, which can compensate for outdated theoretical knowledge in guiding practical decision-making. This result is consistent with a recent global meta-analysis showing that younger healthcare workers display better knowledge, attitudes, and practices regarding antimicrobial resistance than their more senior colleagues, likely reflecting more recent training and greater exposure to updated antimicrobial stewardship curricula [13,16], whereas other studies have instead reported age and years of clinical experience as positive predictors of AMR knowledge [26], a discrepancy that our hospital-wide Italian sample does not support. Taken together, these findings support the need for periodic knowledge-refresher interventions targeted at more senior staff, even when their practical performance appears unaffected, to prevent a widening theory–practice gap as antimicrobial resistance guidelines continue to evolve.

Several methodological limitations should be acknowledged. The limited number of respondents may reduce the generalizability of our results, as well as the heterogeneity among the sub-groups of respondents (e.g., higher participation among nurses than clinicians); in fact, the survey achieved an approximate response rate of 30% in 2024 and 19% in 2025. Although participation declined during the second survey round, response rates in voluntary antimicrobial stewardship surveys vary considerably according to target population and recruitment strategy. Our response rate was higher than that of other reported hospital-based voluntary surveys [27,28], although higher participation has also been described in studies involving more selected professional groups with different recruitment strategies [19,29]. The voluntary survey design introduces potential self-selection bias, which may have led to an overestimation of overall awareness levels at the population level. The inability to link responses across rounds precludes longitudinal analyses at the individual level. In addition, the questionnaire was not formally validated against objective behavioural outcomes, and the generalizability of the findings beyond a single tertiary care hospital may therefore be limited [30]. Additionally, the practice domain was assessed using only three items, a narrower scope compared with the knowledge and attitude domains; while this reflects the structure of the original validated ECDC-derived questionnaire, it may limit the granularity with which practice-related behaviours were captured. Despite these constraints, the analytical approach incorporated important strengths, including the large sample size (*n* = 3193), the inclusion of both clinical and non-clinical staff, and above all the use of two complementary scoring systems, binary and partial credit, which served as a sensitivity analysis, strengthening the robustness of the findings and providing a more nuanced understanding of their distribution across domains, population subgroups, and survey rounds. The consistent convergence of results across these two systems further supports the robustness of the primary conclusions. Building on the present findings, future research should address three main directions. First, longitudinal studies with individual-level linkage across survey rounds are needed to disentangle within-subject changes in knowledge, attitudes and practices from cohort effects. Second, the association between training exposure and questionnaire performance should be validated against objective indicators, including antimicrobial consumption (e.g., DDD/100 patient-days, days of therapy), adherence to institutional guidelines, and incidence of healthcare-associated infections and multidrug-resistant organism colonization. Third, extending this survey framework to multiple hospitals with different AMS maturity levels would improve external validity and support benchmarking of educational reach as a quality indicator of hospital AMS programmes.

## 4. Materials and Methods

***Study design, setting and participants:*** A repeated cross-sectional survey was conducted in October 2024 and October 2025 at a large tertiary-care hospital in north-eastern Italy (Padua), which provides highly specialized medical and surgical care. At the time of the surveys, approximately 6500 staff members were employed at the hospital: about 5700 healthcare workers and 800 non-healthcare workers. These figures do not include medical residents, who are not formally employed by the hospital but have access to institutional email accounts and were therefore also eligible to participate. Both surveys were open to all hospital employees, comprising healthcare and non-healthcare workers. Healthcare professionals included physicians, medical residents, non-medical residents, nurses, healthcare technicians, and healthcare assistants. Non-healthcare workers included administrative staff working in areas such as financial and management control, technical services, information systems, procurement, and logistics. The questionnaire was distributed via the institutional email system to all eligible employees with an active institutional email account. Participation was voluntary, consent-based, and anonymous. To maximize participation, including among staff who were on leave or otherwise unavailable at the time of the initial invitation, a reminder email was sent two weeks after the initial invitation. Because responses were anonymous, individual answers could not be linked across the two survey rounds, and the two datasets were therefore analyzed as independent samples. In 2024, the hospital began implementing a multidimensional antimicrobial stewardship approach program, initially in an Internal Medicine ward [31]. The multidisciplinary team was composed of two infectious disease consultants, two hospital pharmacists, two microbiologists, an infection control nurse, and a hygienist, who conducted bi-weekly audits. In 2025, participation in the national training course on Healthcare-Associated Infections coordinated by the Italian National Institute of Health (Istituto Superiore di Sanità, ISS) as part of the Italian National Recovery and Resilience Plan (PNRR; Mission 6—Health, Component 2, Sub-investment 2.2(b)) was offered to all hospital employees. Therefore, we decided to run two survey rounds to assess any changes in knowledge and awareness over time in relation to the training exposure to the institutional training activities on infection prevention and control and antimicrobial resistance.

***Survey instrument:*** Data were collected using a structured questionnaire titled “Knowledge, practices, and attitudes regarding antibiotic use” (Supplementary Material, Table S1). The survey platform was configured to prevent multiple submissions from the same respondent, and questionnaires were saved only upon completion; partially completed questionnaires were not stored in the database. As a result, no incomplete records or missing data were present in the final dataset, and no additional data-cleaning or imputation procedures were required. The duration of each survey was one month from the date of submission. The survey instrument was adapted from a questionnaire available in Italian language developed within a cross-sectional study promoted by the Department of Public Health and Infectious Diseases of Sapienza University of Rome and validated by the European Centre for Disease Prevention and Control (ECDC) [32]. The questionnaire aimed to assess participants’ knowledge, perceptions and practices related to antibiotic use and antimicrobial resistance. Data were collected and managed using REDCap (Research Electronic Data Capture) [33]. The questionnaire comprised two sections. The first collected demographic and professional information (age, years of service, professional role, and hospital ward/unit), and self-reported participation in training activities related to IPC and AMR. Specifically, participants were asked (i) whether they had attended at least one training activity on appropriate antibiotic use in the previous five years (e.g., continuing medical education courses, workplace-based training, or university teaching), and (ii) whether they had attended the ISS national training course “Prevention and control of healthcare-associated infections”. The second section investigated knowledge (7 items), attitudes (5 items), and practices (3 items) related to antibiotic use and AMR. For each item, five response options were provided, including “I don’t know/not within my field of competence”. To quantify individual performance on the questionnaire, two composite scoring systems were developed based on a predefined set of correct answers established by the investigators prior to data analysis. The “I don’t know/Not my competence” response option was treated as an incorrect answer and assigned a score of zero in both systems. The first approach adopted a binary scoring system, assigning a score of 1 for each correct answer and 0 otherwise. The second approach adopted a partial credit scoring system, assigning 1 for a correct answer, 0.5 for a response consistent with the correct direction (e.g., “agree” when “completely agree” was the correct answer), and 0 for all remaining responses. Under both systems, domain-specific scores were computed for knowledge (range 0–7), attitudes (range 0–5), and practices (range 0–3), as well as a total score (range 0–15).

***Statistical analysis:*** Participant characteristics were described for the overall sample and stratified by survey year (2024 vs. 2025) and by prior attendance at an antibiotic-related training course. Categorical variables were reported as absolute frequencies and percentages, while continuous variables were expressed as median and interquartile range (Q1–Q3). Between-group comparisons were performed using the chi-square test for categorical variables and the Mann–Whitney U test for continuous variables, as appropriate. Total and domain-specific scores were compared between survey years (2024 vs. 2025) and between participants who had or had not attended antibiotic-related training using the Mann–Whitney U test. To examine performance across demographic subgroups, participants were stratified into four age categories (≤34, 35–44, 45–54, and ≥55 years), profession (healthcare staff and non-healthcare staff) and four categories of seniority at the tertiary care hospital (≤10, 11–20, 21–30, and >30 years). To identify independent predictors of questionnaire performance, a multivariable linear regression model was fitted with total score as the dependent variable. The following covariates were included a priori: survey year, healthcare profession, prior attendance at antibiotic-related training and seniority at the tertiary care hospital. Collinearity among covariates was assessed using the variance inflation factor (VIF). Results are expressed as regression coefficients (β) with 95% confidence intervals and two-sided *p*-values. Finally, the pre-specified subgroup analysis was performed by repeating the same descriptive and comparative analyses as in the overall cohort, restricted to clinical healthcare professionals. Between-group comparisons were performed using the Mann–Whitney U test for two-group comparisons and the Kruskal–Wallis test for comparisons across age and seniority categories. All statistical analyses were performed using R (version 4.5.0) [34]. A two-sided *p*-value < 0.05 was considered statistically significant.

## 5. Conclusions

This hospital-wide survey identified persistent opportunities for improvement in AMR-related knowledge, attitudes and, most notably, practices across the entire workforce of an Italian tertiary care hospital. Prior participation in antibiotic-related training emerged as the strongest independent factor associated with questionnaire performance, supporting the value of structured and continuous educational initiatives as a core component of hospital antimicrobial stewardship. Although the repeated cross-sectional design precludes causal inference at the individual level, the modest improvement observed among healthcare professionals in the second survey round, alongside a parallel increase in self-reported training exposure, is consistent with a potential cumulative effect of sustained institutional educational activities. These findings suggest that repeated institutional educational activities may contribute to strengthening stewar dship-related awareness and practices over time. Regular hospital-wide assessment of AMR-related knowledge, attitudes and practices may therefore serve as a pragmatic quality indicator to monitor and refine institutional antimicrobial stewardship programmes over time.

## Figures and Tables

**Table 1:** Characteristics of participants.

		Year			Training Course		
Characteristic	Overall N (%) = 3193 (100)	2024 N (%) = 1928 (60.4)	2025 N (%) = 1265 (39.6)	*p*-Value	No N (%) = 1253 (39.2)	Yes N (%) = 1939 (60.8)	*p*-Value
Age				0.13			**<0.001**
≤34	636 (20)	407 (21)	229 (18)		192 (15)	444 (23)	
35–44	645 (20)	383 (20)	262 (21)		218 (17%)	427 (22)	
45–54	851 (27)	520 (27)	331 (26)		360 (29)	491 (25)	
≥55	1051 (33)	614 (32)	437 (35)		478 (38)	573 (30)	
Seniority				0.5			0.072
≤10	1323 (42)	796 (42)	527 (42)		511 (41)	812 (42)	
11–20	560 (18)	344 (18)	216 (17)		200 (16)	360 (19)	
21–30	577 (18)	336 (18)	241 (19)		229 (19)	348 (18)	
>30	693 (22)	430 (23)	263 (21)		297 (24)	396 (21)	
Year							**0.001**
2024	1927 (60)	-	-		801 (64)	1126 (58)	
2025	1265 (40)	-	-		452 (36)	813 (42)	
Training				**0.001**			
No	1253 (39)	801 (42)	452 (36)		-	-	
Yes	1939 (61)	1126 (58)	813 (64)		-	-	
Role				0.4			**<0.001**
Physician	510 (16)	315 (16)	195 (15)		137 (11)	373 (19)	
Other health professional	84 (2.6)	53 (2.8)	31 (2.5)		57 (4.6)	27 (1.4)	
Nurse	1394 (44)	860 (45)	534 (42)		349 (28)	1045 (54)	
Health collaborator	214 (6.7)	136 (7.1)	78 (6.2)		126 (10)	88 (4.5)	
Physician resident	107 (3.4)	64 (3.3)	43 (3.4)		38 (3.0)	69 (3.6)	
Other health resident	19 (0.6)	12 (0.6)	7 (0.6)		8 (0.6)	11 (0.6)	
Administrative	325 (10)	180 (9.3)	145 (11)		296 (24)	29 (1.5)	
Administrative director	15 (0.5)	7 (0.4)	8 (0.6)		15 (1.2)	0 (0)	
Social-Health Operator	402 (13)	233 (12)	169 (13)		145 (12)	257 (13)	
Other	121 (3.8)	66 (3.4)	55 (4.3)		80 (6.4)	40 (2.1)	

*p*-value was calculated with Pearson’s Chi-squared test.

**Table 2:** Dichotomous scoring system.

		Year			Training Course		
	Overall *n* = 3193	2024 *n* = 1928	2025 *n* = 1265	*p*-Value	No *n* = 1253	Yes *n* = 1939	*p*-Value
Score Knowledge (0–7)	2.93 (1.48)	2.92 (1.47)	2.94 (1.49)	0.7	2.53 (1.54)	3.18 (1.38)	**<0.001**
Score Attitude (0–5)	2.15 (1.05)	2.14 (1.05)	2.16 (1.05)	0.6	1.97 (1.09)	2.26 (1.00)	**<0.001**
Score Practice (0–3)	0.68 (0.65)	0.65 (0.61)	0.72 (0.69)	**0.017**	0.51 (0.49)	0.79 (0.77)	**<0.001**
Score Total (0–15)	5.75 (2.25)	5.71 (2.24)	5.81 (2.26)	0.12	5.01 (2.37)	6.23 (2.02)	**<0.001**

Mean (SD), *p*-value was calculated with Wilcoxon rank sum test.

**Table 3:** Multivariable linear regression analysis of factors associated with total questionnaire score.

	Total			Knowledge			Attitudes			Practice		
	Beta	95% CI	*p*-Value	Beta	95% CI	*p*-Value	Beta	95% CI	*p*-Value	Beta	95% CI	*p*-Value
**Year**												
2024	—	—		—	—		—	—		—	—	
2025	0.81	−0.17, 1.8	0.10	0.64	−0.77, 2.0	0.4	0.19	−1.3, 1.7	0.8	2.3	0.40, 4.1	0.017
**Seniority**												
≤10	—	—		—	—		—	—		—	—	
11–20	−1.1	−2.5, 0.21	0.10	−2.6	−4.6, −0.69	0.008	0.63	−1.4, 2.7	0.5	−0.64	−3.2, 1.9	0.6
21–30	−1.1	−2.4, 0.24	0.11	−2.3	−4.2, −0.36	0.020	−0.68	−2.7, 1.3	0.5	0.96	−1.6, 3.5	0.5
>30	−4.5	−5.8, −3.2	<0.001	−6.4	−8.2, −4.5	<0.001	−4.0	−6.0, −2.1	<0.001	−0.89	−3.3, 1.5	0.5
**Training course**												
No	—	—		—	—		—	—		—	—	
Yes	4.8	3.8, 5.8	<0.001	5.0	3.5, 6.4	<0.001	3.6	2.0, 5.2	<0.001	6.5	4.6, 8.5	<0.001
**Profession**												
Healthcare staff	—	—		—	—		—	—		—	—	
Non-Healthcare staff	−12	−13, −11	<0.001	−16	−18, −14	<0.001	−7.3	−9.1, −5.6	<0.001	−10	−12, −7.9	<0.001

Abbreviation: CI = Confidence Interval.

**Table 4:** Dichotomic score of the subgroup “Health staff”.

		Year			Training Course		
	Overall *n* = 2730	2024 *n* = 1673	2025 *n* = 1057	*p*-Value	No *n* = 860	Yes *n* = 1870	*p*-Value
Score Knowledge (0–7)	3.09 (1.41)	3.07 (1.41)	3.12 (1.42)	0.6	2.84 (1.46)	3.21 (1.38)	**<0.001**
Score Attitudes (0–5)	2.22 (1.02)	2.20 (1.02)	2.24 (1.01)	0.3	2.11 (1.05)	2.26 (1.00)	**<0.001**
Score Practice (0–3)	0.75 (0.72)	0.71 (0.69)	0.80 (0.78)	**0.003**	0.64 (0.60)	0.80 (0.75)	**<0.001**
Score Total (0–15)	6.05 (2.09)	5.99 (2.10)	6.16 (2.06)	**0.046**	5.59 (2.17)	6.27 (2.01)	**<0.001**

Mean (SD), *p*-value was calculated with Mann–Whitney U test.

## Data Availability

Data are contained within the article.

## Supplementary Materials

The following supporting information can be downloaded at: https://www.mdpi.com/article/10.3390/antibiotics15080792/s1, Table S1: Questionnaire, Table S2: Percentage of right answer, Table S3: Partial credit scoring system, Table S4: Characteristics subgroup “Health staff” and Table S5: Partial score subgroup “Health staff”.

## Abbreviations

The following abbreviations are used in this manuscript:

AMR	Antimicrobial resistance
AMS	Antimicrobial Stewardship
HAIs	Healthcare-Associated Infections
IPC	Infection Prevention and Control
WHO	World Health Organization
