# Supplementary material for: Knowledge, Practices, and Attitudes Regarding Antimicrobial Resistance and Antibiotic Use Among Hospital Staff: Findings from a Two-Round Cross-Sectional Survey in an Italian Tertiary Care Hospital
Source: Antibiotics (Basel). 2026 Aug 16;15(8):792. doi: 10.3390/antibiotics15080792 (PMC13509401; doi:10.3390/antibiotics15080792)
Supplement: Supplementary file 1 [file antibiotics-15-00792-s001.zip › antibiotics-4454508-supplementary.pdf]

SUPPLEMENTARY MATERIALS

Table S1. Questionnaire

**Knowledge**					
K1: Antibiotic resistance is a real global problem.	Completely disagree	Disagree	Agree	Completely agree	I do not know
K2: Antibiotic resistance is a real problem for the company where I work.	Completely disagree	Disagree	Agree	Completely agree	I do not know
K3: The development of new antibiotics will solve the problem of antibiotic resistance.	Completely disagree	Disagree	Agree	Completely agree	I do not know
K4: There are bacteria that are resistant to all antibiotics.	Completely disagree	Disagree	Agree	Completely agree	I do not know
K5: An effective antibiotic therapy can be found for any infection.	Completely disagree	Disagree	Agree	Completely agree	I do not know
K6: The cause of antibiotic resistance stems from any antibiotic therapy (whether	Completely disagree	Disagree	Agree	Completely agree	I do not know

appropriate or inappropriate).					
K7: Only incorrect antibiotic therapy (regarding the choice of antibiotic, duration, or method of administration) causes antibiotic resistance.	Completely disagree	Disagree	Agree	Completely agree	I do not know
**Attitude**					
A1: The use of antibiotics is free of significant consequences.	Completely disagree	Disagree	Agree	Completely agree	I do not know
A2: In a hospitalized patient with fever, I would always consider the use of antibiotics to be indicated.	Completely disagree	Disagree	Agree	Completely agree	I do not know
A3: In hospitalized patients with fever, the use of broad-spectrum antibiotics should always be prioritized.	Completely disagree	Disagree	Agree	Completely agree	I do not know
A4: The administration of antibiotics must adhere to the prescribed number	Completely disagree	Disagree	Agree	Completely agree	I do not know

of doses and the scheduled interval.					
A5: Skipping a prescribed dose of antibiotics does not cause significant problems.	Completely disagree	Disagree	Agree	Completely agree	I do not know
**Practice**					
P1: In hospitalized patients with suspected infection, I always collect microbiological specimens before initiating antibiotic therapy.	Always	Often	Sometimes	Never	I do not know
P2: Antibiotic therapy can be discontinued 24 hours after fever resolution.	Always	Often	Sometimes	Never	I do not know
P3: I provide patients with information on the appropriate use of antibiotics and on the problem of antimicrobial resistance.	Always	Often	Sometimes	Never	I do not know

Table S2. Percentage of right answer

	Overall	2024 vs 2025			Trained vs Untrained		
**Characteristic**	**Overall**, N (%) = 3193[1]	**2024**, N (%) = 1928[2]	**2025**, N (%) = 1265[2]	**p-value**[3]	**No**, N (%) = 1253[2]	**Yes**, N (%) = 1939[2]	**p-value**[3]
K1: AMR world problem	2,253 / 3,190 (71)	1,333 (69)	920 (73)	**0.033**	765 (61)	1,488 (77)	**<0.001**
K2: AMR local problem	1,628 / 3,189 (51)	971 (50)	657 (52)	0.4	530 (42)	1,098 (57)	**<0.001**
K3: New antibiotics	419 / 3,185 (13)	243 (13)	176 (14)	0.3	163 (13)	256 (13)	>0.9
K4: Bacterial pan-resistance	808 / 3,187 (25)	470 (24)	338 (27)	0.14	230 (18)	578 (30)	**<0.001**
K5: Therapy always efficacy	1,758 / 3,180 (55)	1,076 (56)	682 (54)	0.3	612 (49)	1,146 (59)	**<0.001**
K6: Wrong therapy	981 / 3,184 (31)	604 (31)	377 (30)	0.4	366 (29)	615 (32)	0.2
K7: Posology	1,493 / 3,183 (47)	925 (48)	568 (45)	0.083	500 (40)	993 (51)	**<0.001**
A1: Antibiotics without consequences	1,185 / 3,191 (37)	698 (36)	487 (38)	0.2	407 (32)	778 (40)	**<0.001**
A2: Antibiotics for fever	882 / 3,188 (28)	498 (26)	384 (30)	**0.006**	309 (25)	573 (30)	**0.003**
A3: Large spectrum	1,584 / 3,187 (50)	971 (50)	613 (49)	0.3	549 (44)	1,035 (53)	**<0.001**
A4: Adherence	1,288 / 3,188 (40)	802 (42)	486 (38)	0.074	518 (41)	770 (40)	0.3
A5: Skip dose	1,916 / 3,186 (60)	1,159 (60)	757 (60)	0.8	686 (55)	1,230 (64)	**<0.001**
P1: Pre-antibiotic samples	1,096 / 3,182 (34)	641 (33)	455 (36)	0.11	310 (25)	786 (41)	**<0.001**
P2: Suspension post-24h	402 / 3,188 (13)	234 (12)	168 (13)	0.4	97 (7.8)	305 (16)	**<0.001**
P3: Patient education	667 / 3,188 (21)	381 (20)	286 (23)	0.058	234 (19)	433 (22)	**0.015**

[1]n / N (%),[2]n (%),[3]Pearson's Chi-squared test

Table S3. Partial credit scoring system

		Year			Training course		
	**Overall**<br>N = 3,193	**2024**<br>N = 1,928	**2025**<br>N = 1,265	**p-value**	**No**<br>N = 1,253	**Yes**<br>N = 1,939	**p-value**
Score Knowledge (0-7)	3.58 (1.38)	3.58 (1.36)	3.58 (1.40)	>0.9	3.17 (1.52)	3.85 (1.20)	**<0.001**
Score Attitudes (0-5)	3.10 (1.01)	3.11 (1.00)	3.10 (1.03)	>0.9	2.87 (1.14)	3.26 (0.88)	**<0.001**
Score Practice (0-3)	1.20 (0.84)	1.17 (0.84)	1.24 (0.86)	**0.034**	0.92 (0.87)	1.38 (0.78)	**<0.001**
Score Total (0-15)	7.89 (2.38)	7.87 (2.34)	7.91 (2.43)	0.3	6.95 (2.68)	8.49 (1.92)	**<0.001**

Mean (SD)

Table S4. Characteristics subgroup "Health staff"

		Year			Training course		
**Characteristic**	**Overall**<br>N (%) = 2,730[1]	**2024**<br>N (%) = 1,673[1]	**2025**<br>N (%) = 1,057[1]	**p-value**[2]	**No**<br>**N (%) = 860**[1]	**Yes**<br>**N (%) = 1,870**[1]	**p-value**[2]
Age				0.3			**<0.001**

≤34	568 (21)	366 (22)	202 (19)		143 (17)	425 (23)	
35-44	560 (21)	337 (20)	223 (21)		148 (17)	412 (22)	
45-54	713 (26)	439 (26)	274 (26)		237 (28)	476 (26)	
≥55	880 (32)	528 (32)	352 (33)		327 (38)	553 (30)	
Seniority				0.5			**<0.001**
≤10	1,094 (41)	674 (41)	420 (40)		315 (37)	779 (42)	
11-20	487 (18)	297 (18)	190 (18)		141 (17)	346 (19)	
21-30	492 (18)	290 (18)	202 (19)		153 (18)	339 (18)	
>30	619 (23)	392 (24)	227 (22)		235 (28)	384 (21)	
Training course				**<0.001**			
No	860 (32)	584 (35)	276 (26)		-	-	
Yes	1,870 (68)	1,089 (65)	781 (74)		-	-	
Year							**<0.001**
2024	1,673 (61)	-	-		584 (68)	1,089 (58)	
2025	1,057 (39)	-	-		276 (32)	781 (42)	

Table S5. Partial score subgroup "Health staff"

		Year			Training course		
	**Overall**<br>N = 2,730	**2024**<br>N = 1,673	**2025**<br>N = 1,057	**p-value**[1]	**No**<br>N = 860	**Yes**<br>N = 1,870	**p-value**[1]
Score Knowledge (0-7)	3.75 (1.26)	3.74 (1.26)	3.77 (1.27)	0.6	3.50 (1.38)	3.87 (1.19)	**<0.001**
Score Attitudes (0-5)	3.21 (0.93)	3.20 (0.93)	3.21 (0.93)	0.6	3.08 (1.02)	3.26 (0.88)	**<0.001**
Score Practice (0-3)	1.32 (0.80)	1.29 (0.79)	1.37 (0.81)	**0.004**	1.14 (0.84)	1.40 (0.77)	**<0.001**
Score Total (0-15)	8.28 (2.06)	8.23 (2.06)	8.35 (2.07)	0.063	7.72 (2.28)	8.53 (1.91)	**<0.001**

Mean (SD)
